# Supplementary material for: Cataloging the phylogenetic diversity of human bladder bacterial isolates
Source: Genome Biol. 2024 Mar 21;25:75. doi: 10.1186/s13059-024-03216-8 (PMC10958879; doi:10.1186/s13059-024-03216-8)
Supplement: Supplementary file 1 — Additional file 1: Fig. S1. Phylogenomic tree of 1134 bladder isolated genomes. Fig. S2. Whole-genome ANI comparison of the 196 different GTDB-identified bacterial species in the bladder isolated genomes. Fig. S3. Phylogenomic tree of 169 bacterial species (assigned by GTDB) represented in the bladder genome collection from the symptomatic group. Fig. S4. Phylogenomic tree of 76 bacterial species (assigned by GTDB) represented in the bladder genome collection from the asymptomatic group. Fig. S5. COG function comparisons of bladder isolated genomes with vaginal and gut isolated genomes. Fig. S6. COG functions enriched in the bladder based on COG category. [file 13059_2024_3216_MOESM1_ESM.pdf]

Supplementary material for

Cataloging the phylogenetic diversity of human bladder bacterial isolates

Jingjie Du[1,&], Mark Khemmani[1], Thomas Halverson[1], Adriana Ene[2], Roberto Limeira[3], Lana Tinawi[3], Baylie R. Hochstedler-Kramer[1], Melline Fontes Noronha[1], Catherine Putonti[2,4], Alan J. Wolfe[1,3*]

1 Department of Microbiology & Immunology, Stritch School of Medicine, Loyola University Chicago, Maywood, IL 60153
2 Bioinformatics Program, Loyola University Chicago, Chicago, IL 60660
3 Loyola Genomics Facility, Stritch School of Medicine, Loyola University Chicago, Maywood, IL 60153
4 Department of Biology, Loyola University Chicago, Chicago, IL 60660
& Current address: Division of Nutritional Science, Cornell University, Ithaca, NY 14850
*Corresponding Author: Alan J. Wolfe, PhD
Department of Microbiology and Immunology, Stritch School of Medicine, Loyola University Chicago, Maywood, IL 60153 USA, awolfe@luc.edu

Additional File 1: Supplementary figures

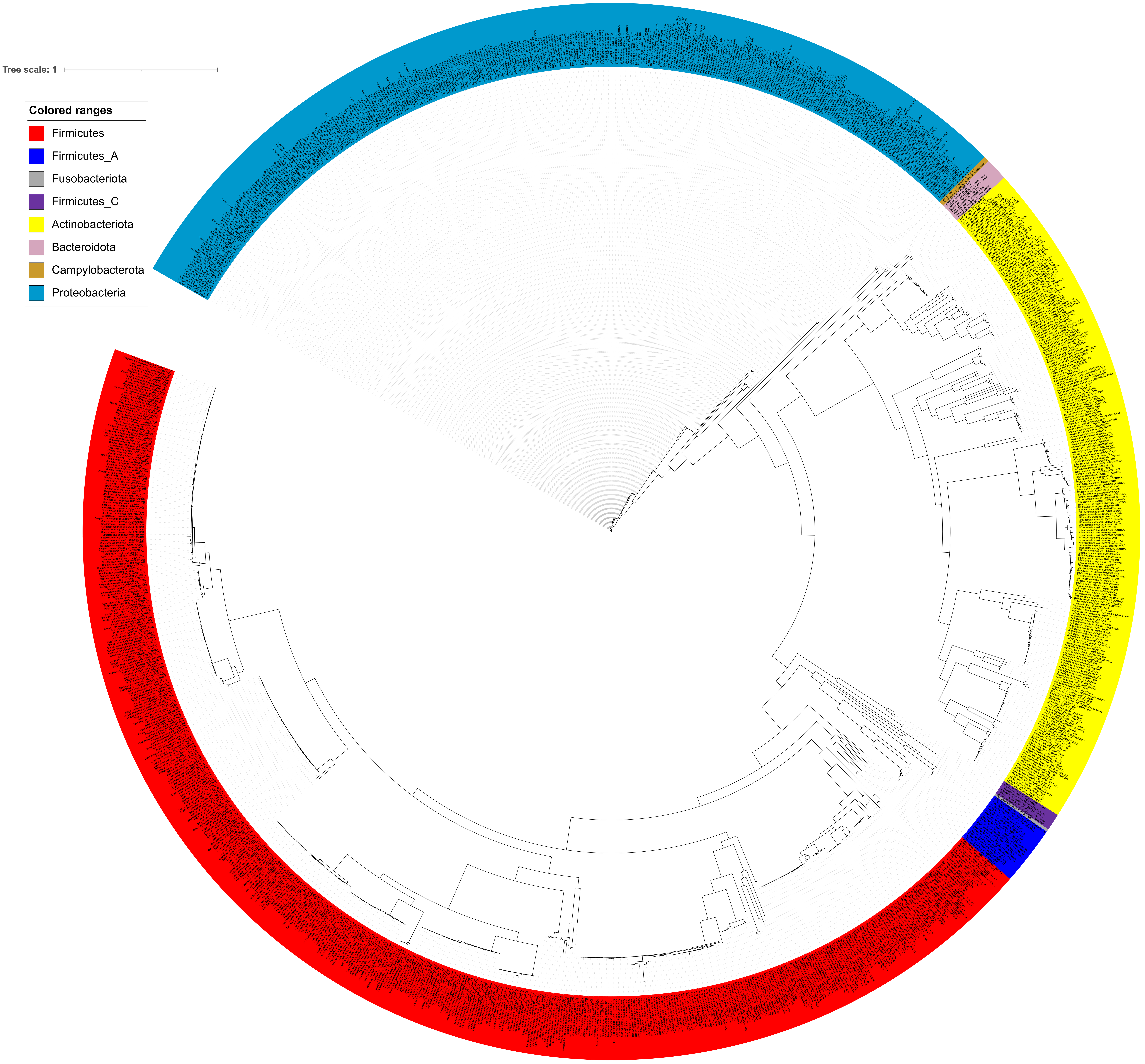

Fig. S1: Phylogenomic tree of 1134 bladder isolated genomes.
71 core universal bacterial genes were used to reconstruct the phylogenetic tree. Bacterial species are colored by GTDB-identified phylum. Disease status of the host for each isolate is indicated in the isolate's name. Additional File 2: Table S1 contains detailed information for the isolates and hosts.

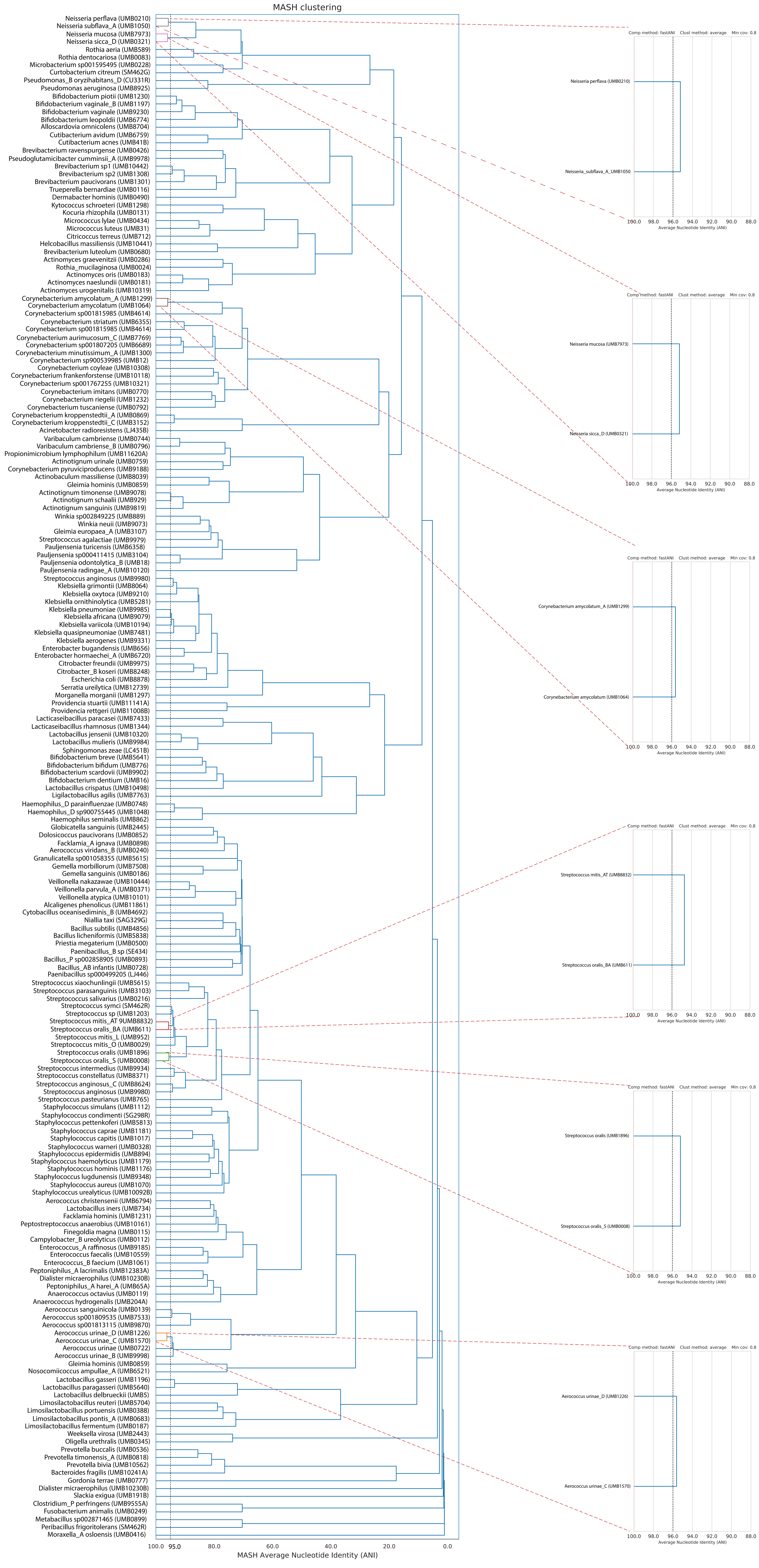

Fig. S2: Whole-genome ANI comparison of the 196 different GTDB-identified bacterial species in the bladder isolated genomes.
The 95% ANI and 96% ANI cut-offs were used to cluster genomes into different groups using MASH clustering and fastANI in dRep.

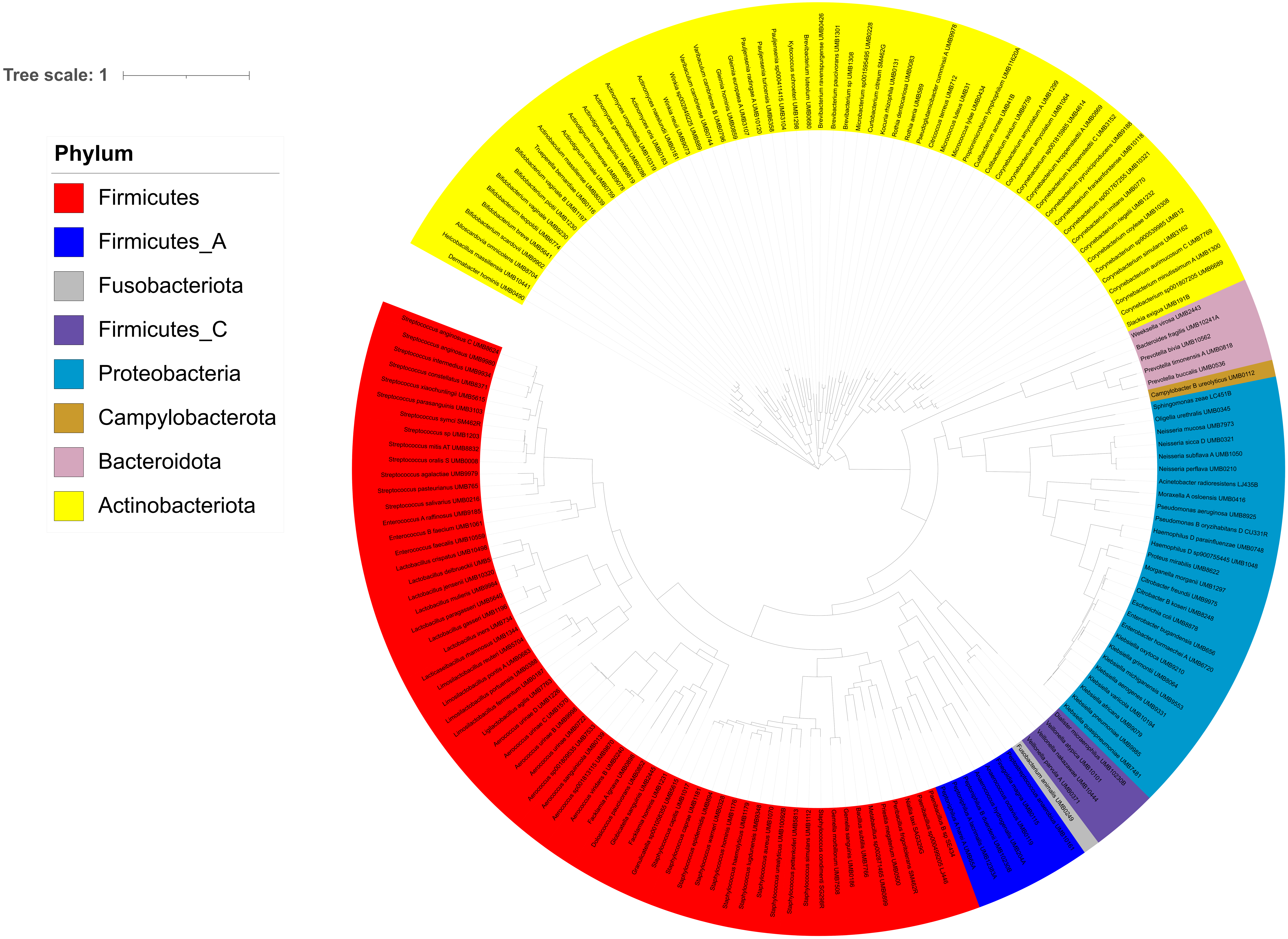

Fig. S3: Phylogenomic tree of 169 bacterial species (assigned by GTDB) represented in the bladder genome collection from the symptomatic group.

One single genome per species was selected out of the 837 high quality isolated genomes (>90% completeness, < 5% contamination) isolated from the symptomatic group to reconstruct the multiple sequence alignment of 71 core bacterial genes. Bacterial species are colored by phylum.

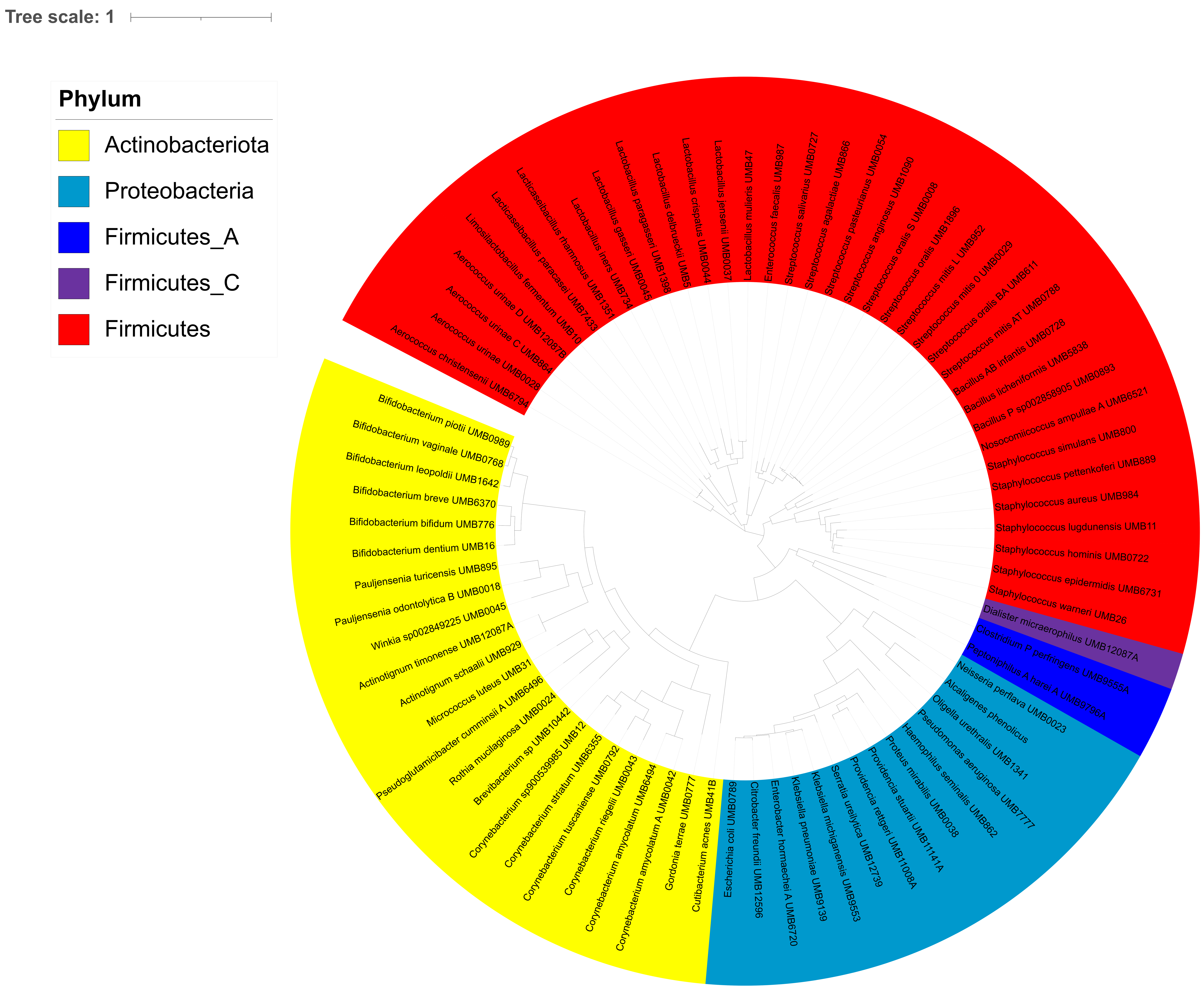

Fig. S4: Phylogenomic tree of 76 bacterial species (assigned by GTDB) represented in the bladder genome collection from the asymptomatic group.

One single genome per species was selected out of the 239 high quality isolated genomes (>90% completeness, < 5% contamination) isolated from healthy group to reconstruct the multiple sequence alignment of 71 core bacterial genes. Bacterial species are colored by phylum.

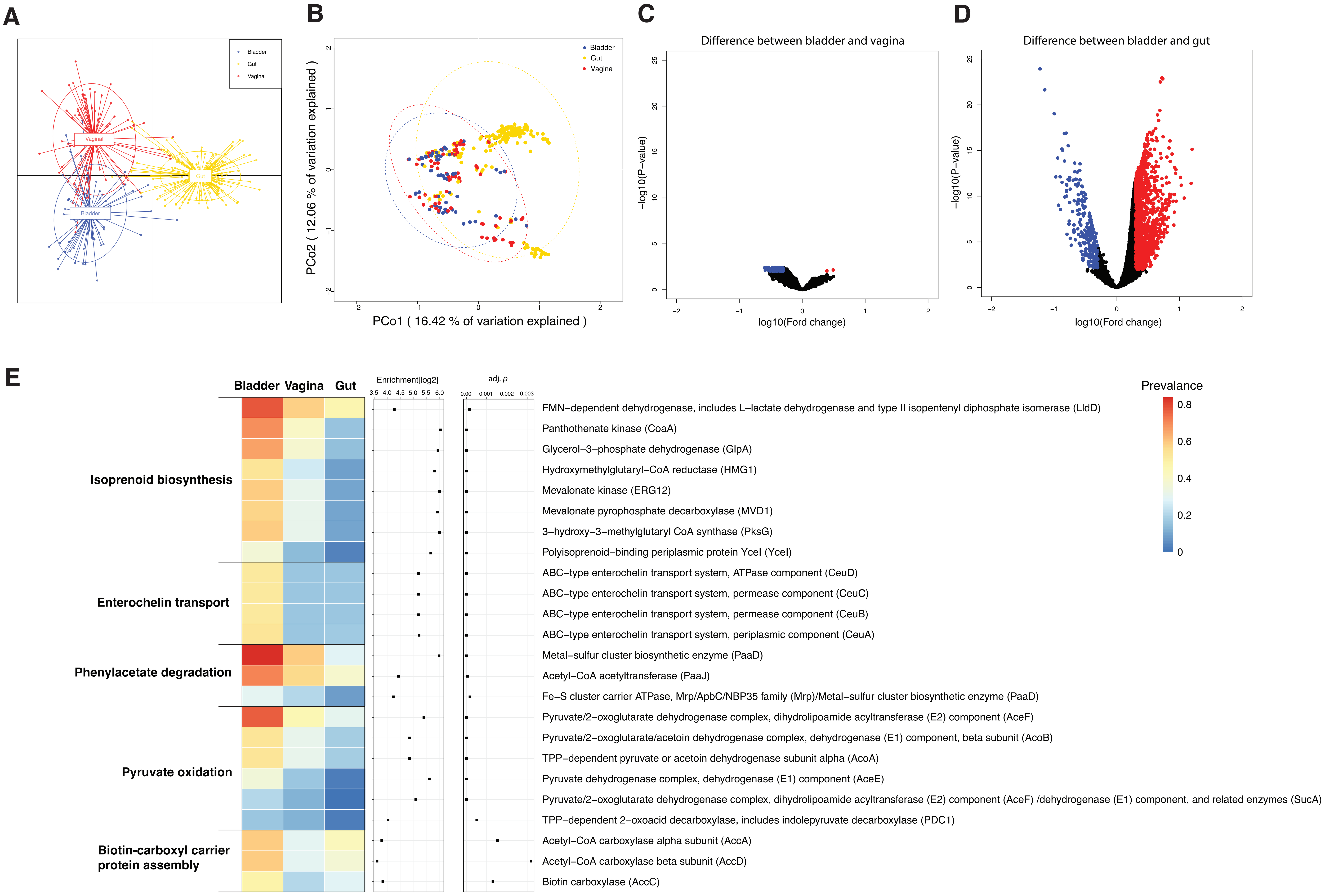

Fig. S5: COG function comparisons of bladder isolated genomes with vaginal and gut isolated genomes.

A-B. DAPC and PCoA of COG functional diversity of bacterial species isolated from the asymptomatic individuals of different niches. **C-D.** Volcano plots of *t* tests corrected by the Benjamini and Hochberg (BH) method for changes of COG functions between bladder and vagina/gut. An FDR cutoff of 0.01 and [logFC] > 0.3 were used. Data points highlighted in red indicate COG functions that were significantly enriched in gut or vagina, while data points highlighted in blue indicate COG functions that were significantly enriched in the bladder. **E.** Selected COG functions that were enriched in the bladder as compared with gut and/or vagina. An FDR cutoff of 0.01 and more than 10% prevalence in all bladder genomes were used.

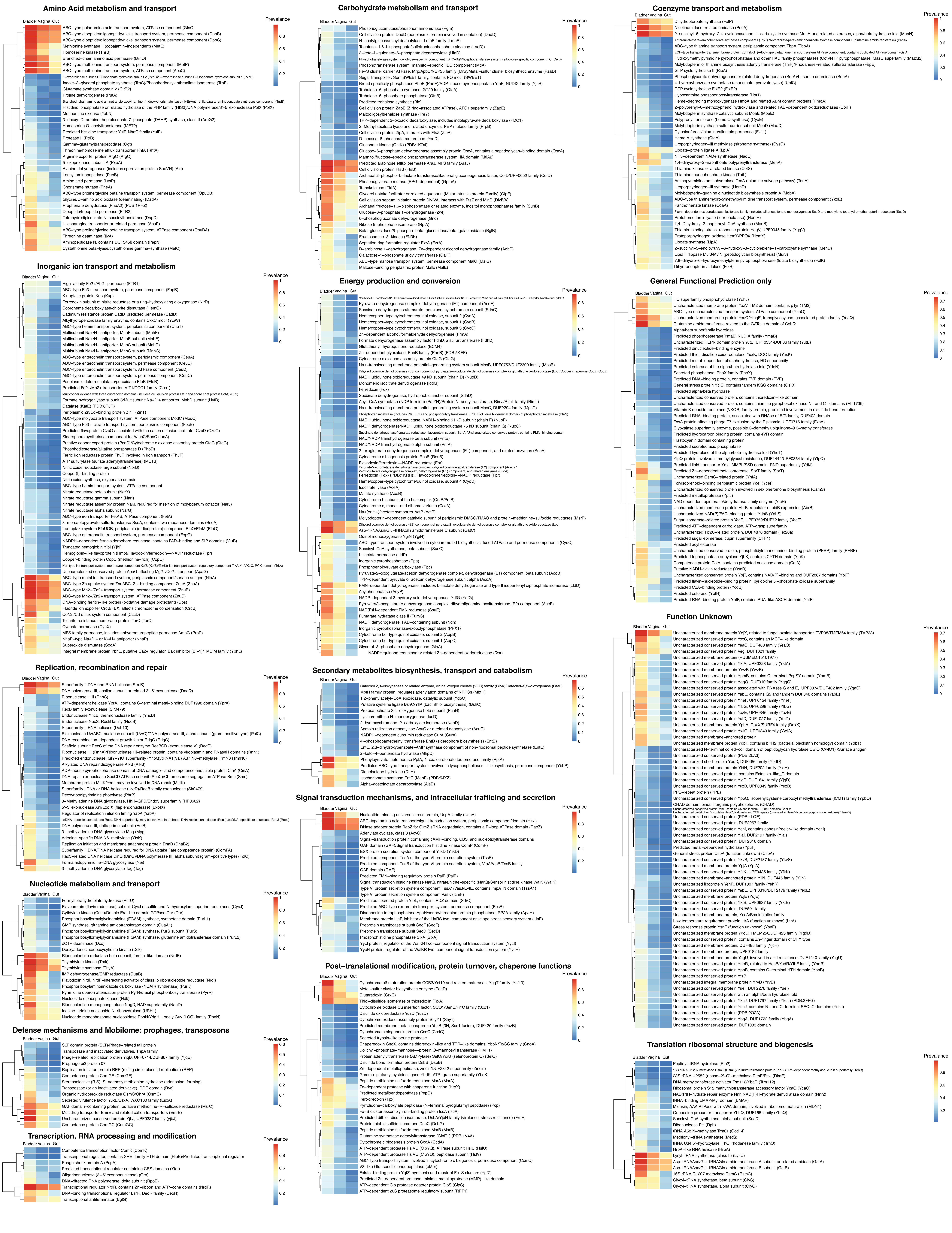

Fig. S6: COG functions enriched in the bladder based on COG category. The COG functions with an FDR cutoff of 0.01 and [logFC] > 0.3 were displayed in different categories.
