# Supplementary material for: Cataloging the phylogenetic diversity of human bladder bacterial isolates
Source: Genome Biol. 2024 Mar 21;25:75. doi: 10.1186/s13059-024-03216-8 (PMC10958879; doi:10.1186/s13059-024-03216-8)
Supplement: Supplementary file 3 — Additional file 3. Review history. [file 13059_2024_3216_MOESM3_ESM.docx]

Review History

**First round of review**

**Reviewer 1**

**Are you able to assess all statistics in the manuscript, including the appropriateness of statistical tests used?** Yes, and I have assessed the statistics in my report.

**Comments to author:**

Du et al. present a catalog of genomes isolated from the human bladder as well as some analyses thereof with a focus on comparing E. coli and showing differences in the function of genes differentially present in the different body site. Overall, this paper is quite straightforward, but summarizes a lot of work and describes a good resource which will indeed be useful to the community. I have comments and suggestions below, which I do think are important to address to make the paper better represent the work done, but I estimate that they can be quickly addressed.

\# Major comments

1. The lack of association between phylogroup membership and symptomatic infection (paragraph L234-L251; and later in the discussion, L332+) needs to be more explicitly tested and some power calculation should be provided. I will note that there are only 11 genomes from asymptomatic controls.

2. Some of the limitations of the study should be more upfront:

2a. The population is almost exclusively female. This is acknowledged in the Strengths and Limitations section, but I would even argue that the title should already reflect this. Alternatively, the authors could provide some data that their collection captures the male population as well.

2b. The study population is from the US or at least "mostly from US participants" (L489), although only US-based institutions are listed. This should acknowledged in the Strengths and Limitations section and earlier on.

3. How exactly were the strains selected (L104+)? I searched the methods and few details are given.

\# Minor comments

The figures are generally clear, but I do worry that they will be unreadable when printed as even their full page versions required very small fonts. Often the main points could be done with significant less detail, while leaving elements such as the full list of strain IDs in 3A for supplemental material. Fig 4C-D are already difficult to read without zooming to 200%.

Fig 3A: The legend can probably be improved by using the actual colours rather than colour names. I would also recommend adding symbols to make the figure more accessible to colourblind individuals.

Fig 4: In my copy, I think what is tagged as Supp Fig 7 is actually Fig 4 and I am reviewing as such.

Fig 4B: Please add "Phylogroup" to the x-axis legend.

\# Suggestions

Abstract and L301: why limit the impact of this resource to "hypothesis-driven research"? I think this would be useful for researchers doing data-driven research as well.

**Reviewer 2**

**Are you able to assess all statistics in the manuscript, including the appropriateness of statistical tests used?** No, I do not feel adequately qualified to assess the statistics.

**Comments to author:**

The authors introduce a substantial addition of bacterial isolates and genomes from the bladder environment. They aim to illustrate the potential of their collection to cover many dominant bladder taxa by comparing it to 16s rRNA sequencing data. Moreover, they compare bladder, gut, and vaginal isolates, suggesting specific similarities and differences. While the resource can be of significant value to the human microbiome community, the way some analyses are presented, and the conclusions drawn raise questions. For this to be considered for publication, the authors must foreground the distinct value of this resource more clearly and ensure their comparisons and conclusions are fully backed by the presented data.

Major Comments

The authors underscore the distinctiveness of their bladder bacterial isolates collection but need to articulate its novelty more explicitly in relation to previous studies. It's vital to elucidate how their collection stands out in terms of depth, breadth, and the novelty of isolates. While they note that earlier research might have conflated urinary and genital microbes, a detailed comparison with these studies would substantiate their claims. Illustrating overlaps, highlighting exclusive isolates to their collection, and delving into the potential implications of these novel strains for bladder health and disease comprehension would make their work more compelling.

In the "The Bladder-specific isolates genomes are diverse and large" section, the authors make a case for the breadth of the collection via 16s rRNA sequencing comparisons. However, using metagenomic data could further strengthen their case. Incorporating findings from the eight bladder shotgun metagenomic sequencing studies they cited could amplify the significance of the presented collection.

In their comparative analysis between bladder, vaginal, and gut isolates, the conclusions seem to overreach what the data can robustly support. If the authors intend to make wide-ranging conclusions about bacterial similarities across body sites, they should consider basing these on broader datasets, such as metagenomic sequencing or more comprehensive 16s rRNA data comparisons.

Exploring the diversity within species or genera in their collection can be valuable. An in-depth look into this aspect could reveal nuances that standard 16s taxonomic evaluations might overlook. Highlighting this aspect would emphasize the depth and granularity of their resource.

Minor Comments

Line 109-110: It is unclear at this point in the text what "symptomatic or asymptomatic individuals" refers to. Based on the next paragraph it seems to be referencing UTI patients, but this should be clarified in these lines.

Line 126: The section title refers to the isolate genomes as being "diverse and large". It is unclear what the genomes being large means based on the text in that section.

Line 181-182: This statement is too broad to make based on only the comparison of small collections of isolates from each environment.

Lines 200-226: Did the authors find any difference in biofilm related genes between the collections? This may be an interesting thing to point out considering the importance of biofilm formation in UTIs development and recurrence.

Lines 504-509: The use of the pangenomes for the enrichment analysis should be mentioned in the results section as well.

**Authors Response**

**Point-by-point responses to the reviewers’ comments:**

We would like to thank the editor and the reviewers for their help in improving our manuscript. We have addressed all of their concerns and have responded positively to most. There are a few, however, that we have chosen to rebut, because the request was either impossible or impractical.

Reviewer reports:

Reviewer #1: Du et al. present a catalog of genomes isolated from the human bladder as well as some analyses thereof with a focus on comparing E. coli and showing differences in the function of genes differentially present in the different body site. Overall, this paper is quite straightforward, but summarizes a lot of work and describes a good resource which will indeed be useful to the community. I have comments and suggestions below, which I do think are important to address to make the paper better represent the work done, but I estimate that they can be quickly addressed.

\# Major comments

1. The lack of association between phylogroup membership and symptomatic infection (paragraph L234-L251; and later in the discussion, L332+) needs to be more explicitly tested and some power calculation should be provided. I will note that there are only 11 genomes from asymptomatic controls.

**Response:** *The analysis performed was with respect to the different symptom groups, with the majority (74%) of the strains being isolated from individuals with UTI symptoms. We acknowledge that there is not sufficient representation of other symptom groups. This limitation in assessing an association between phylogroup membership and symptom status is addressed in the Discussion (lines 342-344):*

“It is important to note that most (74%) bladder *E. coli* isolates were collected from participants diagnosed with UTI or RUTI; future work isolating *E. coli* from the bladders of individuals with the other symptom statuses considered here is needed. “

*We need more representatives of* E. coli *strains isolated from these other symptom groups. It may be that* E. coli *is an infrequent member of the urobiome of individuals with these symptoms. In such a case, individual constituents may not be good indicators of the symptoms.*

*We also acknowledge that there is not sufficient representation of* E. coli *strains from asymptomatic controls. We have added a statement to the Discussion to highlight the need for further sequencing of* E. coli *isolates from asymptomatic controls in an effort to ascertain if phylogroup membership is associated with the lack of symptoms (lines 346-347):*

“Further sequencing of *E. coli* strains from asymptomatic controls, however, is needed as our study only included 11 strains.”

*Finally, we note that other studies that have considered* E. coli *phylogroups and symptomatic vs. asymptomatic participants either have used voided urine or have similarly few representatives from asymptomatic participants.*

2. Some of the limitations of the study should be more upfront:

2a. The population is almost exclusively female. This is acknowledged in the Strengths and Limitations section, but I would even argue that the title should already reflect this. Alternatively, the authors could provide some data that their collection captures the male population as well.

**Response:** *We understand the reviewer’s concern. We thought hard about how to change the title but found no good solution. Instead, in the abstract, we note that the genomes are primarily from adult females (lines 50-51).*

“…here we present a bladder-specific bacterial reference collection comprised of 1134 genomes, primarily from adult females.”

*Likewise, we added this qualification in the Introduction (lines 93-94).*

“In this study, we combined EQUC of urine obtained primarily from adult females by transurethral catheterization….”

2b. The study population is from the US or at least "mostly from US participants" (L489), although only US-based institutions are listed. This should acknowledged in the Strengths and Limitations section and earlier on.

**Response:** *Thanks for noting this omission. We now have acknowledged this limitation in the Strengths and Limitations section (Lines 385-388).*

“Second, the study population mostly consists of US participants. Notably, our previous observations revealed distinct differences in the bladder microbiomes between Chinese and US participants, indicating a possible geographic variation in the bladder microbiome^45^. An ideal collection should include isolates from different countries.”

3. How exactly were the strains selected (L104+)? I searched the methods and few details are given.

**Response:** *Thanks for noting this. We have added details for isolate selection as a separate section in the Methods (Lines 435-441). The section is included below.*

“**Choice of isolates for sequencing**

To assess genomes across as much of the phylogenomic spectrum as possible, we selected at least one member from each species identified by MALDI-TOF for which we had at least one isolate. For some of the species commonly found in either symptomatic or asymptomatic individuals, we chose more than 20 isolates. Given the limited phenotypic or genomic data for most species, the selection process was largely random. For *E. coli*, we chose 186 isolates to ensure that we captured several isolates from each phylogroup.”

\# Minor comments

The figures are generally clear, but I do worry that they will be unreadable when printed as even their full page versions required very small fonts. Often the main points could be done with significant less detail, while leaving elements such as the full list of strain IDs in 3A for supplemental material. Fig 4C-D are already difficult to read without zooming to 200%.

**Response:** *Thanks for the advice. We have revised Figure 3 and Figure 4 to make them more readable. Now, the phylogenetic tree (previously Figure 3A) is now its own Figure 3. The previous Figure 3B, 3C, and 3D are now presented as Figure 4. The previous Figure 4 is now labeled as Figure 5. Note that they are all quite high resolution.*

Fig 3A: The legend can probably be improved by using the actual colours rather than colour names. I would also recommend adding symbols to make the figure more accessible to colourblind individuals.

**Response:** *Thanks for the advice. We have edited the Figure so that the color names are in the actual colors. However, we are not sure how to add symbols to this figure without cluttering it. Note that all the information is Supplemental Table 9, so it is available to our readers.*

Fig 4: In my copy, I think what is tagged as Supp Fig 7 is actually Fig 4 and I am reviewing as such.
**Response:** *Thanks for pointing it out! We are sorry for the mistake and we have corrected it. Now, it’s Figure 5.*

Fig 4B: Please add "Phylogroup" to the x-axis legend.
**Response:** *Thanks for the advice! We have edited the Figure based on the suggestion.*

\# Suggestions

Abstract and L301: why limit the impact of this resource to "hypothesis-driven research"? I think this would be useful for researchers doing data-driven research as well.

**Response:** *Thanks for your advice. In the Abstract, we deleted the phrase “hypothesis-driven” so that the statement is not so specific. At the beginning of the Discussion, we added ‘data-driven’ to the sentence to make it clear that our collection of genomes is a valuable resource for both types of research (Line 301-303).*

*“Here, we have presented the largest collection of bladder-specific bacterial isolates paired with whole-genome sequences, providing a valuable resource for hypothesis-driven* ***and data-driven*** *bladder microbiome research.”*


Reviewer #2: The authors introduce a substantial addition of bacterial isolates and genomes from the bladder environment. They aim to illustrate the potential of their collection to cover many dominant bladder taxa by comparing it to 16s rRNA sequencing data. Moreover, they compare bladder, gut, and vaginal isolates, suggesting specific similarities and differences. While the resource can be of significant value to the human microbiome community, the way some analyses are presented, and the conclusions drawn raise questions. For this to be considered for publication, the authors must foreground the distinct value of this resource more clearly and ensure their comparisons and conclusions are fully backed by the presented data.

Major Comments
The authors underscore the distinctiveness of their bladder bacterial isolates collection but need to articulate its novelty more explicitly in relation to previous studies. It's vital to elucidate how their collection stands out in terms of depth, breadth, and the novelty of isolates. While they note that earlier research might have conflated urinary and genital microbes, a detailed comparison with these studies would substantiate their claims. Illustrating overlaps, highlighting exclusive isolates to their collection, and delving into the potential implications of these novel strains for bladder health and disease comprehension would make their work more compelling.

**Response:** *We appreciate the reviewer’s suggestion. In two places, we had already done this. Near the beginning of the Discussion, we explicitly highlight the difference between the current study and our previously published one, in terms of numbers of genomes and species sequenced and analyzed (lines 314-317).*

“The catalog reported here is a substantial extension of a previously published study^14^. In that previous study, we analyzed 149 bladder genomes from 78 different species. In the current study, we analyzed 1134 genomes from 196 species (**Figure 1E, Supplemental Figure 1**), including 7 species without representative genomes in GTDB or the NCBI taxonomy database.”

*In the Strengths and Limitations section of the Discussion, we explicitly highlight the difference between our studies and those of others in terms of urine collection method (lines 364-369).*

**“**First, we sequenced and analyzed only isolates from urine obtained by transurethral catheterization. Thus, our collection can be considered a bladder-specific bacterial genome reference collection. Most studies of urinary bacterial genomes, regardless of sequencing methodology, have been performed on voided urine. Because voided urine is often contaminated with post-urethral microbes, the origin of each bacterial isolate from a voided urine sample cannot be determined^56^.”

*The point here is NOT that we have sequenced and analyzed genomes from species that are not present in voided urine but RATHER that our readers can be sure that our genomes come from isolates that clearly originated in the bladder. As suggested by the reviewer, we could compare our results to those that sequenced genomes from voided urine and claimed them to be urinary, especially Dubourg et al., 2020 (PMID: 33178140), who claimed that “these results suggest that many members of the microbiota in the urinary tract are in fact derived from the gut.” We choose to not make a direct comparison for two major reasons: 1) the number of differences is huge and 2) we do not want to get into an argument with those authors. We expect that most of our readers will understand that the only way to determine whether a bacterium originated in a particular niche is to sample that niche.*

In the "The Bladder-specific isolates genomes are diverse and large" section, the authors make a case for the breadth of the collection via 16s rRNA sequencing comparisons. However, using metagenomic data could further strengthen their case. Incorporating findings from the eight bladder shotgun metagenomic sequencing studies they cited could amplify the significance of the presented collection.

**Response:** *The eight shotgun metagenomic sequencing studies referenced in the introduction (lines 88-89) are mostly not representative of the bladder microbiome but rather the urogenital microbiome (voided urine). The table below lists the reference and method of urine collection:*

| ***Reference #*** | ***Author*** | ***Method of Collection*** |
| --- | --- | --- |
| 27 | Moustafa et al. 2018 | Void |
| 28 | Garretto et al. 2018 | Catheterization (10 symptomatic and 20 with OAB) |
| 29 | Adebayo et al. 2020 | Void |
| 30 | Janes et al. 2022 | Not specified |
| 31 | Adu-Oppong et al. 2022 | Mixed; 12 were obtained via catheterization (6 from females; 6 from males. Of these samples, 3 were culture-positive, while 9 were culture-negative. |
| 32 | Thänert et al. 2022 | Void |
| 33 | Wehedy et al. 2022 | Void |
| 34 | Neugent et al. 2022 | Void |

*We concluded that there was insufficient shotgun metagenomic sequence data of strictly bladder urine samples. Any trends observed would not have a large enough sample size.*

In their comparative analysis between bladder, vaginal, and gut isolates, the conclusions seem to overreach what the data can robustly support. If the authors intend to make wide-ranging conclusions about bacterial similarities across body sites, they should consider basing these on broader datasets, such as metagenomic sequencing or more comprehensive 16s rRNA data comparisons.

**Response:** *As stated in our response to the previous comment, there are simply not enough shotgun metagenomic sequence data to perform such an analysis. As for the “more comprehensive 16S rRNA data comparisons,” the reviewer has not given us enough information to know precisely what “more comprehensive” means. We argue that the best study design would be to recruit a large number of participants and obtain samples from all 3 sites. Given that it took us more than a decade to build our isolate collection and enough funding to sequence ~1000 genomes, this ideal study has not been performed. We mention this as a limitation (lines 388-391):*

“Third, the bladder, vagina, and gut genomes were obtained from different participants. An ideal design would be to compare the genomes of isolates obtained from all 3 niches from the same cohort of participants. Alas, that collection does not yet exist.”

Exploring the diversity within species or genera in their collection can be valuable. An in-depth look into this aspect could reveal nuances that standard 16s taxonomic evaluations might overlook. Highlighting this aspect would emphasize the depth and granularity of their resource.

**Response:** *We agree. In the Strengths and Limitations section, we have added a statement to that effect (lines 371-375).*

“Third, whole genome sequencing provides a collection of high-quality genomes for the phylogenetic diversity found within the bladder, permitting exploration beyond what can be achieved by 16S rRNA gene sequencing. For example, we used some of these genomes to determine that the species Aerococcus urinae is actually a complex of at least 4 species and 2 groups^20^.”

This is a resource manuscript. The value of the resource should be better stated in the manuscript.

**Response: *We make this point at the end of the abstract*** *(lines 61-63):*

*“*This bladder-specific bacterial reference collection is a unique resource that will enable bladder microbiota research and comparison to isolates from other anatomical sites.”

***and at the beginning of the Discussion (Lines 301-303):***

“Here, we have presented the largest collection of bladder-specific bacterial isolates paired with whole-genome sequences, providing a valuable resource for hypothesis-driven and data-driven bladder microbiome research.”

***We consider this to be sufficient.***

Minor Comments
Line 109-110: It is unclear at this point in the text what "symptomatic or asymptomatic individuals" refers to. Based on the next paragraph it seems to be referencing UTI patients, but this should be clarified in these lines.

**Response:** *We do not understand the reviewer’s problem with this phrase. It simply refers to species that are commonly found in catheterized urine samples obtained from individuals who either do or don’t have symptoms. It means that we did not only sequence a lot of genomes from typical uropathogens but also typical commensals. The rest of the paragraph lists those species.*

 Line 126: The section title refers to the isolate genomes as being "diverse and large". It is unclear what the genomes being large means based on the text in that section.

**Response:** *We agree. The header, as written, is unclear. We have simply deleted “and large.”*

Line 181-182: This statement is too broad to make based on only the comparison of small collections of isolates from each environment.

**Response:** *We have softened our conclusion. It now reads as follows (lines 182-184):*

“Taken together, these results are consistent with the hypothesis that bacterial species from each niche are distinct, and that the bladder shares more bacterial species with the vagina than the gut.”

Lines 200-226: Did the authors find any difference in biofilm related genes between the collections? This may be an interesting thing to point out considering the importance of biofilm formation in UTIs development and recurrence.

**Response:** *We appreciate the reviewer’s suggestion. However, we have chosen to not do so. There are two reasons. First, we were not exploring the differences between genomes from UTI-positive and UTI-negative individuals. Second, in the interest of space, we had to make choices and thus focused on those functions that were most enriched. However, note that these enriched functions included genes associated with capsule, adhesion, and flagella, which are all involved in biofilm formation. Remember that a major purpose of the study was to provide high quality genomes from isolates known to originate in the bladder for future genome mining studies. An in-depth exploration of biofilm-associated genes is something that researchers can now pursue with confidence that the genomes are from bladder isolates.*

Lines 504-509: The use of the pangenomes for the enrichment analysis should be mentioned in the results section as well.

**Response:** Agreed. *We now make the following statement in the Results (line 185).*

“We next used the pangenomes to evaluate functional differences in the microbiota….”

**Second round of review**

**Reviewer 1**

The authors have addressed all my concerns.

**Reviewer 2**

The authors present a revised manuscript on their human bladder isolate collection. The author's revisions to the manuscript have helped address some of the reviewers' concerns, especially regarding some of the methodology and study limitations. However, the authors have chosen not to perform any additional analyses to address any concerns regarding comparisons to other studies or datasets. There is no doubt that this isolate/genome collection is a valuable resource and helps to fill in a gap in an understudied portion of the human microbiome, but the original concerns about how the data is presented and the conclusions that are drawn remain. Without some comparison to other studies, it remains difficult to assess the authors’ statements about the novelty and utility of their resource.

The authors chose not to perform a comparison to a relevant study, Dubourg et al., 2020, because ": 1) the number of differences is huge and 2) we do not want to get into an argument with those authors.". Considering that one of the main points of the study appears to be that this is a novel, bladder-specific collection, it would seem that providing some degree of comparison to other studies would be warranted. The authors state that the number of differences between their study and the previous voided urine studies is huge, which highlights the value of the resource their presenting. However, very few details are given to show that the studies are different, limiting the impact of the author's statements in the manuscript.

Thank you for providing additional details related to the previously conducted metagenomic studies. Based on the author's counts from the table provided in the response, at least 42 metagenomes were obtained via catheterization in these previous studies. This would appear to be a reasonable amount of samples to use in your study as a comparison, highlighting the utility of your presented genome collection. Is there a reason why these samples obtained via catheterization would not be suitable for these purposes?

**Authors Response**

**Point-by-point responses to the reviewers’ comments:**

*We thank the editor and reviewers for their efforts. We believe our response should be satisfactory.*

Reviewer #2: 
The authors chose not to perform a comparison to a relevant study, Dubourg et al., 2020, because ": 1) the number of differences is huge and 2) we do not want to get into an argument with those authors.".  Considering that one of the main points of the study appears to be that this is a novel, bladder-specific collection, it would seem that providing some degree of comparison to other studies would be warranted. The authors state that the number of differences between their study and the previous voided urine studies is huge, which highlights the value of the resource their presenting. However, very few details are given to show that the studies are different, limiting the impact of the author's statements in the manuscript. 

*Response: We have re-written the beginning of the Strengths and Limitations section emphasizing the limitations of voided urine and specifically mentioning the Dubourg study (lines 377-387). It now reads as follows:*

This study has several strengths. First, we sequenced and analyzed only isolates from urine obtained by transurethral catheterization, which is known to sample urine from the bladder^10, 61^. In contrast, most studies attempting to inventory bacterial species of the urinary tract have been performed on voided urine. A prime example is a recent study by Dubourg and colleagues^62^, who used their own enhanced culture method to detect microbes in voided urine. These authors claimed to have increased the microbial repertoire of the urinary tract and concluded that many urinary species originate from the gut; however, they should have restricted their conclusions to urine and not the urinary tract. This is because voided urine is often contaminated with post-urethral microbes, and thus the origin of each bacterial isolate from a voided urine sample cannot be determined^56^. In contrast, because we sequenced only isolates from catheterized urine, our collection can be considered a bladder-specific bacterial genome reference catalog.

*We have chosen to not perform a detailed comparison of our catalog to that of Dubourg and of others because the differences are huge(numbering in the hundreds of species) and the problem with using a catalog obtained from voided urine is obvious – the origin of the isolates is unknown. The strength of our work is that the public can be sure that the isolates are of bladder origin.*

Thank you for providing additional details related to the previously conducted metagenomic studies. Based on the author's counts from the table provided in the response, at least 42 metagenomes were obtained via catheterization in these previous studies. This would appear to be a reasonable amount of samples to use in your study as a comparison, highlighting the utility of your presented genome collection. Is there a reason why these samples obtained via catheterization would not be suitable for these purposes?

*Response: We have now performed this analysis and we obtain results (77.8%) similar to those of the 16S comparison (72.2%). The text now reads as follows (lines 157-182):*

We also analyzed publicly available metagenomes from 42 urine samples collected using catheters across two studies^28, 31^. Our sequenced genomes represented 77.8% (21/27) of the bacterial genera and 52.8% (28/53) of the bacterial species (**Supplemental Table 3**).

Among the 50 genera detected by either 16S rRNA gene sequencing or metagenomics sequencing, 12 genera lack strains within our genome collection: *Agathobacter*, *Anaeroglobus*, *S5-A14a* (a genus in Anaerovoracaceae family) *Sneathia*, *Fannyhessea*, *Magasphaera*, *Mobiluncus*, *Porphyromonas*, *Ureaplasma*, *Ezakiella*, *Jonquetella*, and *Lawsonella*. These genera are strictly anaerobic, particularly fastidious, or lack a cell wall.

For other abundant anaerobes detected by either 16S sequencing or metagenomics sequencing, such as *Anaerococcus* and *Prevotella*, we had only a few strains in our cultured isolate collection (**Supplementary Figure 1, Supplementary Table 1**). For example, the average relative abundance of *Anaerococcus* and *Prevotella* is about 2.8% and 3.2%, respectively, among all 16S rRNA gene sequenced samples, but our collection of 5619 isolates only contains 3 *Anaerococcus* and 5 *Prevotella* strains. Moreover, our sequenced genomes represented only a few of the *Anaerococcus* and *Prevotella* species detected by metagenomic sequencing (**Supplementary Table 3**).

In contrast, we were able to isolate and thus sequence taxa that were simply missed by 16S rRNA gene or metagenomics sequencing or were present only as rare taxa (<0.1% average relative abundances across all individuals); these included isolates from the genera *Acinetobacter*, *Alcaligenes*, *Bacillus*, *Bacteroides, Citricoccus, Citrobacter*, *Curtobacterium*, *Cytobacillus, Dermabacter, Dolosicoccus, Enterobacter*, *Gemella*, *Gleimia*, *Globicatella*, *Gordonia*, *Granulicatella*, *Haemophilus*, *Helcobacillus*, *Kocuria*, *Kytococcus*, *Ligilactobacillus*, *Metabacillus*, *Microbacterium*, *Moraxella_A*, *Morganella*, *Micrococcus*, *Neisseria*, *Niallia*, *Nosocomiicoccus*, *Paenibacillus*, *Pauljensenia*, *Peribacillus*, *Priestia*, *Rothia*, *Serratia*, *Slackia*, *Sphingomonas*, *Trueperella*, *Weeksella* and *Oligella* (**Supplementary Tables 1, 2 and 3**).

*Associated methods are now included (lines 564-577).*
